# Chinese residents’ knowledge about and behavior towards dairy products: a cross-sectional study

**DOI:** 10.1186/s12889-023-15254-1

**Published:** 2023-02-21

**Authors:** Jun Wei, Jian Wang

**Affiliations:** 1 Songnan Town Community Health Service Center, 301 Songliang Road, Baoshan District, Shanghai, 200441 China; 2 Department of General Practice, International Medical Center, Zhongshan Hospital, Fudan University, 130 Yixueyuan Road, Shanghai, 200032 China

**Keywords:** Dairy product, Knowledge, Behavior, Residents

## Abstract

**Background:**

Chinese residents generally had insufficient intake of dairy products. The correct mastery of dairy related knowledge helps to develop a good habit of dairy intake. Dairy intake and purchasing behavior were related to multiple factors. As an attempt to lay a scientific basis for guiding the rational intake of dairy by Chinese residents, we launched a survey to discover Chinese residents’ knowledge about dairy product, intake and purchasing behavior of dairy and its influencing factors.

**Methods:**

An online survey was conducted from May to June 2021, and 2500 Chinese residents aged 16–65 were selected using the convenient sampling method. A self-designed questionnaire was adopted. Analysis of the demographic and sociological factors influencing Chinese residents' knowledge about dairy products, behavior towards dairy intake and purchasing were measured.

**Results:**

The average score of knowledge about dairy product of Chinese residents was 4.13 ± 1.50 points. 99.7% of the respondents considered that drinking milk was beneficial, whereas only 12.8% gained a correct insight into the specific benefits of drinking milk. 4.6% of the respondents correctly knew what nutrients they could get from milk. 4.0% of the respondents could correctly identify the type of dairy product. 50.5% of the respondents knew that adult should drink at least 300 ml milk per day. Female, young and high-income residents had better dairy knowledge, while residents with lactose intolerance and whose family members do not have a milk-drinking habit had worse dairy knowledge (*P* < 0.05). On average, Chinese residents consumed 255.61 ± 88.40 ml dairy product per day. The elderly residents, residents with low education level, residents who lived with family members without milk-drinking habits and residents with poor knowledge of dairy product had worse dairy intake behavior (*P* < 0.05). When purchasing dairy products, young and middle-aged people (54.20% of those aged ≤ 30, 58.97% of those aged 31–44, and 57.08% of those aged 45–59) were most concerned about whether probiotics were added to dairy products. The elderly (47.25%) were most concerned about whether dairy products were low sugar / sugar free. Chinese residents (52.24%) tended to purchase small-packaged dairy products that could be consumed easily anytime and anywhere.

**Conclusion:**

Chinese residents had a lack of knowledge regarding dairy products, and their dairy intake was insufficient. We should further strengthen the popularization of dairy product related knowledge, guide residents to choose dairy products correctly, and increase the intake of dairy products by Chinese residents.

**Supplementary Information:**

The online version contains supplementary material available at 10.1186/s12889-023-15254-1.

## Background

Dairy is a type of food with high nutritional value. The common dairy products on the market consist of milk, yogurt, cheese, milk powder, etc. As a good source of high-quality protein, dairy provides the whole set of exogenous amino acids required by the synthesis of body protein. Dairy also contains fat, lactose, and multiple vitamins and minerals [1]. Besides providing nutrients, fermented dairy products such as yogurt are also rich in probiotics [2–5]. Numerous national dietary guidelines include dairy as a recommended food. To be specific, the Dietary Guideline of Australia recommended 750 ml, the Dietary Guideline of South Africa recommended 250 ml, and the Chinese Dietary Guideline recommended at least 300 ml milk for adults per day [1, 6].

As revealed by existing studies, the dairy intake of residents aged 18–44 in nine provinces in China in 2006 was 11.45 ml per day [7]. In 2011, the dairy intake of residents aged 18–59 in nine provinces reached 25.71 ml per day [8]. In 2015, the dairy intake of residents aged 18–59 in 15 provinces was 20.67 ml per day [9]. In 2019, China's per capita dairy purchase quantity reached 93.33 ml per day [10]. The per capita dairy purchase quantity was not an accurate intake quantity, but it generally showed positive correlation with intake quantity. Although per capita dairy purchase quantity had been rising on a year-to-year basis, dairy intake of Chinese residents remained at a low level, which had not reached the recommended standard of China's Dietary Guideline. In 2020, the outbreak of the COVID-19 pandemic had significantly changed people's lifestyles and dietary habits. Has the consumption behavior towards dairy, a recommended food, changed accordingly in China at present?

It has been demonstrated that the correct grasp of nutrition knowledge is conductive to the development of healthy dietary behavior [11]. The purchasing behavior of dairy products is related to many factors. Philip Kotler, a prominent economist, had proposed that purchasing behavior was mainly influenced by cultural, social, personal and psychological factors [12]. Therefore, the analysis of the correlation between these factors and dairy purchasing behavior could provide some references for improving Chinese residents' dairy intake behavior. Hence, we conducted an online survey of Chinese residents, aiming to discover their knowledge about and behavior towards dairy products and their influencing factors, as an attempt to lay a scientific basis for guiding Chinese residents' rational intake of dairy products.

## Methods

### Participants

The cross-sectional survey was conducted from February to March 2021. 2,500 Chinese residents aged 16–65 were selected as participants by the convenient sampling method. The samples were sourced from 20 cities in China (including Beijing, Tianjin, Shenyang, Shanghai, Nanjing, Hangzhou, Zhengzhou, Wuhan, Changsha, Guangzhou, Shenzhen, Chongqing, Chengdu, Xi’an, Harbin, Qingdao, Xiamen, Hohhot, Taiyuan, as well as Nanning). The respondents filled in an online questionnaire via a link established on Dataway Horizon's platform. A total of 2500 questionnaires were collected, and 21 invalid questionnaires (data missing) were excluded, with the effective recovery rate of 99.16%.

### Data collection

A self-designed questionnaire was used. The questionnaire included the following information: (1) Demographic information: gender, age, education level, region, annual income; (2) Whether the respondent was lactose intolerant; (3) Milk drinking habits of family members; (4) 10 choice questions of dairy knowledge, with question 9,11,13,14,16,17 as single choice questions, question 8,10,12,15 as multiple choice questions. For each single choice question, 1 point for correct choice. For each multiple-choice question, if all the correct answers are selected, 1 point will be counted. The total score is 0–10. Respondents with scores higher than 60% of all respondents were defined as having good mastery of dairy [13]. (5) Dairy intake and purchasing behavior: Gap filling questions such as daily intake of dairy product, days of dairy intake per week, and years of dairy intake. Choice questions such as “how often you check ingredient statement on the label when buying dairy products”, problems such as the demand for information about dairy products, the information that people were concerned about when buying dairy products, and the possible additional consumption pattern of dairy products the paticipant would like to try in the near future.

### Ethical approval

This protocol was approved by the Ethics Committee of Wusong Central Hospital, Baoshan District, Shanghai (2021-Y-09) and all the participants gave informed consent to participate in the study before taking part.

### Statistical analysis

SPSS 20.0 was used for data processing and analysis. Percentage and median were used to describe the basic characteristics of demography and the correct answer rate. Measurement data were expressed as mean(X) ± standard deviation(S). T test was used for comparison of measurement data between two groups, and F test was used for comparison between multiple groups. Multiple response analysis was used to describe the multiple-choice response distribution. Chi-square test was used as a univariate analysis to compare the differences in knowledge mastery of dairy products among different groups. Multivariate logistic regression analysis was conducted to analyze the influencing factors of dairy knowledge mastery. All statistical analysis was conducted by two-sided test, and *P* < 0.05 was considered statistically significant.

## Results

### Basic information

A total of 2,479 people were analyzed. There were 1,203 women and 1,276 men. The respondents ranged from 16 to 65 years in age, with a mean age of 37.30 ± 11.97 years (Table 1).

**Table 1:** Basic information of respondents

Variable		Number	Proportion (%)
Gender	male	1276	51.47
	female	1203	48.53
Age	≤ 30 years old	882	35.58
	31–45 years old	814	32.84
	45–59 years old	692	27.91
	≥ 60 years	91	3.67
Education level	below undergraduate	1178	47.52
	Bachelor’s degree or above	1301	52.48
Place of residence	City	2224	89.71
	County	198	7.99
	Countryside	57	2.30
Annual income	< 50,000RMB	597	24.08
	50,000–150,000RMB	1496	60.35
	> 150,000RMB	386	15.57
Do you have lactose intolerance?	Yes	348	14.04
	No	2044	82.45
	unknown	87	3.51
Do other family members (including children over 3 years old) who live with you drink milk?	All	877	35.38
	Part	1528	61.64
	None	41	1.65
	Living alone	33	1.33

### Mastery of knowledge of dairy products

For the knowledge of dairy products, the lowest score was 0, the highest score was 10, the median score was 4, and the average score was 4.13 ± 1.50 for Chinese residents. 4.0% of the respondents could correctly identify the type of dairy products, and the lowest recognition rate was butter (28.4%). 99.7% of the respondents considered that drinking milk was beneficial, 84.8% considered that people should drink milk every day. 43.9% correctly knew who should drink milk every day, and only 12.8% knew the specific benefits of drinking milk. 4.6% of the respondents correctly knew what nutrients they could get from milk. 50.5% of the respondents knew that adult should intake at least 300 ml of dairy products per day. 3.1% of the respondents knew that adults should intake at least 800 mg of calcium a day. (Table S1). [see Additional file 1].

According to single-factor analysis, women, young residents, highly educated residents, urban residents, residents with high income, residents without lactose intolerance, and residents who lived with family members with milk-drinking habits had a good mastery of dairy knowledge (*P* < 0.05) (Table 2).

**Table 2:** Single-factor analysis of residents' knowledge about dairy products

Variable	Category	Good mastery of knowledge		Chi-square	Significance
		Number	Percentage		
Gender	male	483	37.9%	4.940	0.026
	female	508	42.2%		
Age	≤ 30 years old	354	40.1%	22.857	0.000
	31–45 years old	371	45.6%		
	45–59 years old	238	34.4%		
	≥ 60 years	28	30.8%		
Education level	below undergraduate	425	36.1%	14.211	0.000
	Bachelor degree or above	566	43.5%		
Place of Residence	City	906	40.7%	6.733	0.035
	County	62	31.3%		
	Countryside	23	40.4%		
Annual income	< 50,000RMB	194	32.5%	23.519	0.000
	50,000–150,000RMB	614	41.0%		
	> 150,000RMB	183	47.4%		
Do you have lactose intolerance?	Yes	106	30.5%	22.627	0.000
	No	861	42.1%		
	unknown	24	27.6%		
Do other family members (including children over 3 years old) who live with you drink milk?	All	424	48.3%	49.229	0.000
	Part	549	35.9%		
	None	5	12.2%		
	Living alone	13	39.4%		

Multi-factor logistic regression analysis showed that women, young residents and residents with high income had a good mastery of dairy knowledge, while residents with lactose intolerance and who lived with family members without milk-drinking habits had a poor mastery of dairy knowledge (Table 3).

**Table 3:** Multi-factor analysis of residents' knowledge about dairy products

Variable		B	Significance	OR	95% CI	
					Lower limit	Upper limit
Gender		0.207	0.015	1.230	1.042	1.452
Age		-0.145	0.015	0.865	0.770	0.972
Education level		0.039	0.705	1.040	0.849	1.275
Place of Residence		-0.143	0.192	0.867	0.699	1.074
Annual income		0.353	0.000	1.423	1.231	1.646
Do you have lactose intolerance?			0.000			
	Yes	-0.091	0.740	0.913	0.534	1.562
	No	0.465	0.063	1.593	0.974	2.603
Do other family members (including children over 3 years old) who live with you drink milk?			0.000			
	All	0.377	0.307	1.458	0.707	3.003
	Part	-0.121	0.741	0.886	0.432	1.817
	None	-1.352	0.025	0.259	0.079	0.845

### Dairy intake behavior

On average, Chinese residents consumed 255.61 ± 88.40 ml of dairy product per day, 254.38 ± 91.04 ml for men and 256.89 ± 85.56 ml for women. T test and F test showed that compared with men, women consumed dairy products for more days per week (*P* < 0.05) and persisted in consuming dairy products for longer years (*P* < 0.05). Some people were old, or poorly educated, or did not always check dairy products’ ingredient labels, or their family members didn’t habitually drink milk, or had little knowledge about dairy products. They showed worse performance in the number of days they consume dairy products each week, number of years they keep consuming dairy products, and the daily intake amount of dairy products (*P* < 0.05). (Table S2). [see Additional file 2]

### Diary purchasing behavior

36.55% of Chinese residents checked ingredient statement on the label when buying dairy products, 46.15% checked the label most of the time, 15.69% checked the label occasionally, and only 1.61% never checked the label. Multiple response analysis showed that the three most important indicators for Chinese residents to purchase dairy products were “probiotics, such as active lactic acid bacteria was added” (56.15%), “low fat/defat” (50.02%), and “low sugar/no sugar”(47.27%). The information that Chinese residents wanted the most to know about dairy products was “nutrition composition and function of dairy products”(69.46%). When asked “Which of the following possible additional consumption pattern of dairy products would you like to try in the near future?”, 52.24% of Chinese residents selected “dairy products in small packages that can be eaten anytime and anywhere” as the top choice. The elderly were less likely to check the labels when purchasing dairy products. The proportion of the elderly who "occasionally" checked the labels (26.37%) and "never" checked the labels (5.49%) were higher than that of young and middle-aged residents. When purchasing dairy products, young and middle-aged people (54.20% of those aged ≤ 30, 58.97% of those aged 31-44, and 57.08% of those aged 45-59) were most concerned about whether probiotics were added to dairy products. The elderly (47.25%) were most concerned about whether dairy products were low sugar / sugar free. (Table S3). [see Additional file 3]

## Discussion

We found that the score of dairy knowledge among Chinese residents was only 4.13 ± 1.50, and only 4.0% of them could correctly identify different kinds of dairy products. There are many kinds of dairy products. Dairy products such as yogurt, milk powder, cheese and butter can be choices for residents who do not like to drink fresh milk. For instance, cheese contains more than 20% protein. It contains six to eight times as much calcium as fresh milk. The content of B Vitamins, Vitamin A and Vitamin D in cheese is several times higher than fresh milk. Butter has a unique flavor and is rich in fat. It provides essential fatty acids which facilitates the absorption of fat-soluble vitamins. However, butter had the lowest recognition rate in this survey, accounting for 28.4%. Therefore, strengthening the publicity of dairy products and helping residents to improve their awareness of different types of dairy products such as cheese and butter will provide more choices for residents to buy dairy products.

Dairy is rich in a variety of high-quality protein, fat, vitamins and micronutrients, and it is an important source of dietary calcium [1]. The optimal calcium intake for Chinese residents aged 14–17 years old and over 50 years old is 1000 mg/d, and that for people aged 18–49 years old is 800 mg/d [14]. Dairy is an important source of calcium for Chinese people, with a calcium content of 322 mg per 300 ml liquid milk. In this study, half of the people (50.5%) knew that adults should take in at least 300 ml liquid milk per day on average [1], 4.6% knew what kind of nutrition they could get from dairy product, and 3.1% knew that adults should take in 800 mg calcium per day [14], thus indicating that most people lack knowledge about the nutritional composition of milk. Calcium is one of the essential micronutrients that play an important role in keeping bones and teeth healthy. However, insufficient calcium intake is common among adults in many countries [15, 16]. Chinese residents are also chronically deficient in calcium [17]. Therefore, improving residents' knowledge about dairy nutritional components and dietary calcium will help people understand the importance of dairy product and dietary calcium, so as to increase the intake of dairy products.

While emphasizing the need to improve calcium deficiency in Chinese residents by promoting the intake of dairy products, we should also pay attention to the adverse effects resulting from the excessive intake of dairy products. The World Health Organization (WHO) has found the coexistence of high calcium intake and high incidence of osteoporosis in some countries, which is called calcium paradox [18]. Residents in these countries consume more milk. Calcium content in milk is about three times and phosphorus content in milk is about six times that of human milk. When you drink milk, you can absorb most of the phosphorus but only about a third of the calcium. When consumed in large quantities, dairy products and other phosphorus-containing foods can affect the balance of calcium and phosphorus in the serum, triggering the release of calcium from the bones by parathyroid hormones and thus increasing the risk of osteoporosis [19]. The phosphorus content per 300 ml of milk is about 268 mg, which is safe for Chinese residents as recommended by Chinese Dietary Guideline. While encouraging residents to drink milk, publicity should also be carried out to let residents know the appropriate intake amount of various nutritional elements in dairy products and the harm of excessive intake, so that Chinese residents can consume diary healthily and safely.

Vitamin D is closely related to the absorption and metabolism of calcium and phosphorus in intestine [20]. Long-term deficiency of vitamin D may lead to a variety of diseases [21–23]. Multiple studies have shown that vitamin D supplementation can reduce the risk of osteoporosis [19]. Diary is a natural source of vitamin D. Cheese contains three to six times as much vitamin D as in fresh milk among a variety of dairy products, serving as a better source of Vitamine D for people. Chinese youngsters, pregnant women and elderly people are generally deficient in vitamin D [24–26]. We recommend that these people eat dairy products with higher vitamin D content, such as cheese, to alleviate their vitamin D deficiency.

Sodium is one of the body's essential electrolytes. Dairy products contain sodium, and cheese is high in sodium. It is acknowledged that high sodium diet results in increased risk of cardiovascular disease [27]. A study on older adults’ insensitivity to salt revealed that high-cheese diet, despite its high sodium content, could protect vascular endothelial function by limiting oxidative stress [28]. Whether cheese consumption has the same effect in other populations deserves further study.

Dairy products also contain magnesium. Magnesium is an essential element involved in the normal life activities and metabolism of the human body. Studies have shown that appropriate supplementation of magnesium can improve vascular endothelial function [29] and reduce blood pressure [30, 31]. Milk contains about 33 mg of magnesium per 300 ml and people can choose dairy products as one of the sources of dietary magnesium.

This survey showed that the vast majority of respondents (99.7%) considered that drinking milk was beneficial, and 84.8% considered that they should drink milk every day, revealing that Chinese residents had realized the importance of drinking milk and had a good sense of drinking milk. Unfortunately, only 43.9% of the respondents correctly identified who should drink milk every day, and 12.8% correctly identified the specific benefits of drinking milk. Milk can provide a variety of nutrients for human body. Fermented dairy products play an important role in protecting oral cavity [2] and intestinal health [4] because they are rich in probiotics. Therefore, dairy is recommended for people of all ages in many countries in the world [6]. Only by making people fully aware of the various benefits of dairy to human body and correctly knowing who are suitable to drink milk, can we promote people's behavior of eating dairy products correctly.

We found that compared with the elderly, young group had a better mastery of dairy knowledge. This survey was an online survey, and the respondents were mainly young and middle-aged people who often used the internet. Since young people have more access to more information channels and receive more information regarding dairy, they master more dairy-related knowledge. This may probably be the reason for the knowledge differences between different age groups.

It was reported that women had better knowledge about nutrition than men [32]. In our study, women also had a better grasp of dairy knowledge than men, which might be associated with the fact that women pay more attention to health.

In our survey, high-income groups had a better grasp of dairy knowledge. Income was often positively correlated with education level [33, 34]. The higher the education level, the easier people gain nutritional knowledge correctly. Our finding was consistent with the research of Wang S et al. [35].

We found that lactose intolerant group had a poor mastery of dairy knowledge. When people with lactose intolerant drink milk containing a certain amount of lactose, they will undergo abdominal discomfort, and these discomfort symptoms often lead them to avoid eating dairy products. However, this group of people can choose yogurt or low-lactose dairy products such as low-lactose milk, yogurt and cheese to increase nutrition intake. Furthermore, they can also check ingredient statement on the label for lactose levels, avoid drinking milk on an empty stomach, drinking milk in small amounts and multiple times or drinking milk with other grain foods [1]. For people with lactose intolerant, strengthening the popularization of dairy knowledge and guiding them to choose dairy products correctly is recognized as the entry point to improve their dairy intake behavior.

Our survey indicated that residents whose family members had no habit of drinking milk had a worse mastery of dairy knowledge. The study of Drywień M et al. showed that nutrition intake behavior was affected by family environment [36]. A correct mastery of nutrition knowledge would contribute to the formation of healthy dietary behaviors [11]. Strengthening the publicity of dairy knowledge for family members, improving the level of family dairy knowledge, and exerting the positive influence of family on personal eating behavior will be conducive to promoting the dairy intake behavior of Chinese residents.

This study showed that the average daily intake of dairy product of the respondents was 255.61 ± 88.40 ml, and there’s still a gap between it and the recommended daily consumption of at least 300 ml of milk in the Chinese Dietary Guideline. However, compared with the per capita dairy purchase quantity of 93.33 ml/d in 2019 [10], it had made significant progress. Further analysis revealed that elderly residents, residents with low education level, residents who did not check ingredient statement on the label every time they bought dairy products, residents who lived with family members without the habit of drinking milk, and residents who had poor knowledge of dairy products had worse performance in dairy intake behavior. The study of Ni Mhurchu C et al. reported that the use of nutrition labels might make consumers buy healthier food [37]. People paying attention to product labels and nutrition information had healthier eating habits [38, 39]. In our study, 36.55% of the respondents checked ingredient statement on the label every time they bought dairy products. Those checking the labels every time performed better in the days of consuming dairy products per week, the years of consuming dairy products and the daily intake amount of dairy products, thus confirming the positive role of the use of nutrition labels in healthy diet. At present, there is no relevant research on consumers checking nutrition statement on the label when purchasing dairy products. As revealed by existing studies, proper mastery of nutritional knowledge could significantly improve residents' diet behavior [40–42]. In this study, residents with poor dairy-related knowledge performed worse in dairy intake behavior, which also proved the above point of view. People should be guided to pay attention to the nutrition statement label of dairy products, and residents should be encouraged to check nutrition statement on the label when purchasing dairy products, so as to improve the correct dairy knowledge level, which will be conducive to boosting the improvement of dairy intake behavior. Our survey also revealed that the elderly were less likely to check the labels when purchasing dairy products. We should design dairy product labels that are more convenient for the elderly to read and understand, so as to provide an effective approach for them to learn more about dairy products through the nutrition labels and choose more suitable dairy products for themselves.

Our research suggested that the elderly residents had poor dairy intake behavior. For the elderly, dairy intake can increase the intake of protein and calcium and prevent them from sarcopenia and osteoporosis, which is of high significance to their health [43]. Improving the nutrition knowledge level contributes to the formation of healthy dietary behaviors [11]. We can strengthen the popularization of dairy-related knowledge for the elderly, as an attempt to improve their daiy intake behavior.

According to our survey, the information about dairy products the respondents wanted to know most was “nutrition composition and function of dairy products” (69.5%). Through effective popularization of science and health education, people are bound to improve their knowledge about dairy products.

54.3% of the respondents were most concerned about whether probiotics were added in dairy products, which contributes to human gastrointestinal health. In 2017, the World Gastroenterology Organisation (WGO) pointed out that probiotics could effectively prevent and treat gastrointestinal diseases [44]. In 2018, the European Society for Primary Care Gastroenterology (ESPCG) stated that probiotics could significantly relieve or improve symptoms of lower gastrointestinal tract, such as abdominal pain, abdominal distension, aerofluxus and constipation [45]. We recommend that people with gastrointestinal symptoms take dairy products added with probiotics. Developing more probiotics species suitable to be added into dairy products is also one of the research focuses.

Whether the dairy products were low fat/defat (48.4%) was also of concern to respondents. Saturated fatty acids were the main type of fatty acids found in dairy fats [46]. Studies had revealed positive correlation between total saturated fat intake and risk of death [47]. However, the correlation between saturated fat and cardiovascular disease remained controversial [47]. There was also no definite evidence that full-fat dairy products intake would increase the risk of cardiovascular disease [48, 49]. Dietary guidelines in some countries recommend consumption of low-fat dairy products. WHO firstly recommended the consumption of low-fat dairy products in 2018, and Canada's Food Guideline discouraged consumption of high-fat dairy products in 2019. At present, the Chinese Dietary Guideline have not made guiding recommendations on low-fat dairy products [1]. In-depth study on different saturated fatty acids will contribute to a more comprehensive understanding of the function of dairy fat, so as to provide a reference for Chinese residents on how to reasonably intake dairy products.

The top concern among the elderly was whether dairy products were "low sugar/no sugar". Dairy products contains lactose. Lactose in natural dairy products is not high, such as pure cow milk contains about 4.5–5% of lactose. However, in the manufacturing process of some dairy products, sugar is added in order to elevate the palatability. Excessive intake of sugar can be a risk factor for obesity and metabolic syndrome [50]. In recent years, people have become more aware of the adverse effects of excessive intake of sugar. For people with the demand to control their weight or people with metabolic syndrome, natural dairy products with low sugar content, such as pure milk, may be a healthier option.

When answering “Which of the following possible additional consumption pattern of dairy products would you like to try in the near future?”, the answer “small packages of dairy products that can be eaten anytime and anywhere” was the most selected (52.2%) by respondents. With the accelerated life rhythm, instant food has gradually become a fast nutritional source favored by people, which indicating that increasing the convenience of the consumption of dairy products should be one of the factors to be considered in the development of dairy products. In recent years, the single life has gradually become popular, and small packaged food is welcomed by single consumer groups benefitting from the light size, convenient storage and the availability to determine the amount based on individual needs to avoid waste. Developing small packaged dairy products for this consumer group will be a good choice.

There are some limitations in this study. For instance, due to the limited sampling method, the online survey could merely represent a limited group. The questionnaire would be dependent on retrospective self-report of dietary behavior, which may cause deviations from the actual situation. To effectively improve the dairy-eating behavior of Chinese residents, more research should be carried out in depth to provide strong evidence.

## Conclusions

To sum up, Chinese residents still lack knowledge about dairy nutrition and have insufficient dairy intake. Strengthening the popularization of dairy knowledge and guiding residents to choose dairy products correctly are the entry points to improve the dairy consumption behavior of Chinese residents.

## Supplementary Information

**Additional file 1:** **Table S1.** Dairy knowledge of the respondents.

**Additional file 2:** **Table S2.** Analysis of dairy intake behavior of Respondents.

**Additional file 3:** **Table S3.** Diary purchasing behavior of Chinese residents.

**Additional file 4.** Questionnaire on public milk drinking.

## Data Availability

The data sets used and/or analyzed during the current study are available from the corresponding author on reasonable request.
