# Supplementary material for: Chinese residents’ knowledge about and behavior towards dairy products: a cross-sectional study
Source: BMC Public Health. 2023 Feb 21;23:374. doi: 10.1186/s12889-023-15254-1 (PMC9943042; doi:10.1186/s12889-023-15254-1)
Supplement: Supplementary file 1 — Additional file 1: Table S1. Dairy knowledge of the respondents. [file 12889_2023_15254_MOESM1_ESM.docx]

**Table S1 Dairy knowledge of the respondents**

| **Questions** | **Choice** | **Frequency** | **Percentage（%）** | **Number of respondents who answered correctly** | **Percentage（%）** |
| --- | --- | --- | --- | --- | --- |
| 1.Which of the following do you think is dairy? | (1) Milk | 1613 | 65.1 | 100 | 4.0 |
|  | (2) Yogurt | 1835 | 74.0 |  |  |
|  | (3) Milk powder | 1701 | 68.6 |  |  |
|  | (4) Condensed milk | 1670 | 67.4 |  |  |
|  | (5) Cream | 1680 | 67.8 |  |  |
|  | (6) Cheese | 2048 | 82.6 |  |  |
|  | 1. Food made of milk/milk powder as the main ingredient, such as milk flakes | 1716 | 69.2 |  |  |
|  | (8) Butter | 704 | 28.4 |  |  |
| 2.Which of the following is more consistent with your understanding of milk? | (1) Milk is a nutrient used to replenish the body | 1554 | 62.7 | 925 | 37.3 |
|  | 1. Milk is a daily necessity food as well as vegetables and fruits | 925 | 37.3 |  |  |
| 3. What nutrients do you think you can get from milk? | (1) Protein | 2265 | 91.4 | 113 | 4.6 |
|  | (2) Carbohydrates | 1017 | 41.0 |  |  |
|  | (3) the calcium | 2159 | 87.1 |  |  |
|  | (4) Fat | 1123 | 45.3 |  |  |
|  | (5) Vitamin | 1100 | 44.4 |  |  |
|  | (6) inorganic salt | 421 | 17.0 |  |  |
| 4.Do you think drinking milk is beneficial? | (1) Very beneficial | 2072 | 83.6 | 2471 | 99.7 |
|  | (2) There are some benefits | 399 | 16.1 |  |  |
|  | (3) No benefits | 8 | 0.3 |  |  |
| 5.What do you think are the benefits of drinking milk? | (1) Improve immunity | 2226 | 89.8 | 318 | 12.8 |
|  | (2) Prevention of osteoporosis | 2161 | 87.2 |  |  |
|  | (3) Replenish energy | 1854 | 74.8 |  |  |
|  | (4) Calm the mind | 1071 | 43.2 |  |  |
|  | (5) Improve eyesight | 520 | 21.0 |  |  |
| 6.What kind of dairy is recommended for people with lactose intolerance? | (1) Fresh milk | 507 | 20.5 | 1796 | 72.5 |
|  | (2) Yogurt | 431 | 17.4 |  |  |
|  | (3) Milk powder | 84 | 3.4 |  |  |
|  | (4) Lactose free or low lactose dairy products | 1365 | 55.1 |  |  |
|  | (5)Can not eat any dairy products | 92 | 3.7 |  |  |
| 7.Do you think people should drink milk every day? | (1) Yes | 2102 | 84.8 | 2102 | 84.8 |
|  | (2) No | 151 | 6.1 |  |  |
|  | (3) It doesn't matter | 226 | 9.1 |  |  |
| 8.Which of the following people do you think should drink milk every day? | (1) infants | 2260 | 91.2 | 1088 | 43.9 |
|  | (2) children | 2311 | 93.2 |  |  |
|  | (3) teenagers | 2107 | 85.0 |  |  |
|  | (4) young people | 1702 | 68.7 |  |  |
|  | (5) middle-aged people | 1442 | 58.2 |  |  |
|  | (6) The elderly | 1898 | 76.6 |  |  |
| 9. How much liquid milk do you think adult should drink per day on average? | (1)100ml | 55 | 2.2 | 1251 | 50.5 |
|  | (2)200ml | 1173 | 47.3 |  |  |
|  | (3)300ml | 947 | 38.2 |  |  |
|  | (4)400ml | 162 | 6.5 |  |  |
|  | (5)500ml | 142 | 5.7 |  |  |
| 10.At least how much calcium do you think an adult should take per day? | (1)400mg | 604 | 24.4 | 76 | 3.1 |
|  | (2)500mg | 1112 | 44.9 |  |  |
|  | (3)600mg | 542 | 21.9 |  |  |
|  | (4)700mg | 145 | 5.8 |  |  |
|  | (5)800mg | 76 | 3.1 |  |  |
