# Supplementary material for: Chinese residents’ knowledge about and behavior towards dairy products: a cross-sectional study
Source: BMC Public Health. 2023 Feb 21;23:374. doi: 10.1186/s12889-023-15254-1 (PMC9943042; doi:10.1186/s12889-023-15254-1)
Supplement: Supplementary file 2 — Additional file 2: Table S2. Analysis of dairy intake behavior of Respondents. [file 12889_2023_15254_MOESM2_ESM.docx]

**Table S2 Analysis of dairy intake behavior of Respondents**

|  |  | Days of dairy intake per week | | | | Years of dairy intake | | | | Dairy intake per day (ml) | | | |
| --- | --- | --- | --- | --- | --- | --- | --- | --- | --- | --- | --- | --- | --- |
|  |  | Average | Standard deviation | T/F | Significance | Average | Standard deviation | T/F | Significance | Average | Standard deviation | T/F | Significance |
| Gender | male | 4.223 | 2.326 | -2.013 | 0.044 | 7.780 | 4.612 | -3.445 | 0.001 | 254.384 | 91.043 | -0.669 | 0.504 |
|  | female | 4.411 | 2.325 |  |  | 8.499 | 5.188 |  |  | 256.890 | 85.562 |  |  |
| Age | ≤ 30 years old | 4.398 | 2.184 | 7.743 | 0.000 | 8.808 | 5.569 | 11.391 | 0.000 | 262.739 | 94.922 | 4.029 | 0.007 |
|  | 31-45 years old | 4.512 | 2.318 |  |  | 8.128 | 4.875 |  |  | 255.573 | 92.649 |  |  |
|  | 45-59 years old | 4.059 | 2.427 |  |  | 7.314 | 3.937 |  |  | 247.894 | 74.54 |  |  |
|  | ≥60 years | 3.637 | 2.726 |  |  | 7.394 | 3.535 |  |  | 240.211 | 66.292 |  |  |
| Education level | below undergraduate | 4.099 | 2.397 | -4.386 | 0.000 | 7.560 | 4.554 | -5.184 | 0.000 | 247.801 | 79.980 | -3.938 | 0.000 |
|  | Bachelor’s degree or above | 4.510 | 2.245 |  |  | 8.627 | 5.157 |  |  | 262.389 | 94.625 |  |  |
| Place of Residence | city | 4.327 | 2.329 | 0.515 | 0.598 | 8.164 | 4.896 | 0.793 | 0.452 | 255.502 | 87.970 | 0.055 | 0.947 |
|  | county | 4.253 | 2.330 |  |  | 7.693 | 4.879 |  |  | 255.608 | 91.559 |  |  |
|  | countryside | 4.035 | 2.252 |  |  | 8.346 | 5.691 |  |  | 259.615 | 95.506 |  |  |
| Annual income | ＜50,000RMB | 4.166 | 2.233 | 4.184 | 0.015 | 8.261 | 5.138 | 2.676 | 0.069 | 256.401 | 87.394 | 2.843 | 0.058 |
|  | 50,000-150,000RMB | 4.300 | 2.353 |  |  | 7.954 | 4.884 |  |  | 252.735 | 86.438 |  |  |
|  | >150,000RMB | 4.601 | 2.348 |  |  | 8.605 | 4.639 |  |  | 265.335 | 96.535 |  |  |
| Do you have lactose intolerance? | Yes | 4.276 | 2.314 | 8.468 | 0.000 | 8.058 | 4.974 | 0.788 | 0.455 | 246.049 | 96.188 | 5.512 | 0.004 |
|  | No | 4.364 | 2.326 |  |  | 8.170 | 4.903 |  |  | 258.194 | 86.974 |  |  |
|  | unknown | 3.322 | 2.213 |  |  | 7.452 | 4.950 |  |  | 230.685 | 84.283 |  |  |
| Do other family members (including children over 3 years old) who live with you drink milk? | All | 5.048 | 1.942 | 56.739 | 0.000 | 8.794 | 5.057 | 12.709 | 0.000 | 268.095 | 90.763 | 12.097 | 0.000 |
|  | Part | 3.970 | 2.412 |  |  | 7.760 | 4.739 |  |  | 248.759 | 85.686 |  |  |
|  | None | 2.195 | 2.136 |  |  | 4.690 | 3.175 |  |  | 211.655 | 85.320 |  |  |
|  | Living alone | 3.424 | 2.385 |  |  | 8.000 | 6.469 |  |  | 225.000 | 87.665 |  |  |
| Do you check ingredient statement on the label | Every time | 4.659 | 2.325 | 18.557 | 0.000 | 8.539 | 4.975 | 3.276 | 0.020 | 263.259 | 93.441 | 5.758 | 0.001 |
|  | Most of the time | 4.289 | 2.255 |  |  | 7.846 | 4.743 |  |  | 254.380 | 87.069 |  |  |
|  | Occasionally | 3.645 | 2.366 |  |  | 8.075 | 5.187 |  |  | 239.890 | 77.054 |  |  |
|  | Never | 3.750 | 2.529 |  |  | 7.471 | 5.247 |  |  | 262.647 | 89.182 |  |  |
| Do you have a good mastery of dairy knowledge? | No | 3.868 | 2.283 | -12.115 | 0.000 | 7.782 | 4.874 | -4.012 | 0.000 | 240.540 | 82.186 | -9.588 | 0.000 |
|  | Yes | 4.986 | 2.230 |  |  | 8.628 | 4.929 |  |  | 277.048 | 92.460 |  |  |
