# Supplementary material for: Chinese residents’ knowledge about and behavior towards dairy products: a cross-sectional study
Source: BMC Public Health. 2023 Feb 21;23:374. doi: 10.1186/s12889-023-15254-1 (PMC9943042; doi:10.1186/s12889-023-15254-1)
Supplement: Supplementary file 3 — Additional file 3: Table S3. Diary purchasing behavior of Chinese residents. [file 12889_2023_15254_MOESM3_ESM.docx]

**Table S3 Diary purchasing behavior of Chinese residents**

| **Questions** | **Choice** | **F****requency of residents with different age (percentage%)** | | | | **Total frequency(percentage%)** |
| --- | --- | --- | --- | --- | --- | --- |
|  |  | ≤30 | 31-44 | 45-59 | ≥60 |  |
| Do you check ingredient statement on the label when you buy dairy products? | Every time | 328(37.19) | 285(35.01) | 266(38.38) | 27(29.67) | 906(36.55) |
|  | Most of the time | 399(45.24) | 390(47.91) | 320(46.17) | 35(38.46) | 1144(46.15) |
|  | Occasionally | 138(15.65) | 131(16.09) | 96(13.85) | 24(26.37) | 389(15.69) |
|  | Never | 17(1.93) | 8(0.98) | 10(1.44) | 5(5.49) | 40(1.61) |
| In general, what do you value most when buying dairy products?  （You can select only 3 items） | Low fat/no fat | 475（53.85） | 395（48.52） | 329（47.54） | 41（45.05） | 1240（50.02） |
|  | Low glucose/no sugar | 418（47.40） | 393（48.28） | 318（45.95） | 43（47.25） | 1172（47.27） |
|  | High fiber | 153（17.35） | 108（13.27） | 111（16.02） | 14（15.39） | 386（15.57） |
|  | Added probiotics such as active lactic acid bacteria | 478（54.20） | 480（58.97） | 395（57.08） | 39（42.86） | 1392（56.15） |
|  | Added minerals such as calcium, iron, zinc and selenium | 316（35.83） | 296（36.36） | 242（34.92） | 28（30.77） | 882（35.57） |
|  | Added vitamins | 189（21.43） | 170（20.89） | 143（20.64） | 17（18.68） | 519（20.93） |
|  | Added lactoferrin/whey protein | 241（27.32） | 198（24.32） | 131（18.90） | 13（14.29） | 583（23.51） |
| What do you want to know about dairy products  （You can select only 2 items） | nutrition composition and function of dairy products | 627（71.09） | 563（69.17） | 468（67.53） | 64（70.33） | 1722（69.46） |
|  | Production process and food safety control of dairy products | 405（45.92） | 412（50.61） | 333（48.05） | 39（42.86） | 1189（47.96） |
|  | How to eat dairy products and combine them with other meals | 307（34.81） | 222（27.27） | 217（31.31） | 28（30.77） | 774（31.21） |
|  | How to make food from dairy products, such as homemade milk tea | 130（14.74） | 93（11.43） | 95（13.71） | 9（9.89） | 327（13.19） |
|  | How to choose the appropriate dairy products | 185（20.98） | 193（23.71） | 161（23.23） | 22（24.18） | 561（22.62） |
|  | How to solve problems related to dairy intake, such as lactose intolerance | 47（5.33） | 66（8.11） | 38（5.48） | 9（9.89） | 160（6.45） |
| Which of the following possible additional consumption pattern of dairy products would you like to try in the near future？  （You can select only 2 items） | dairy products in small packages that can be eaten anytime and anywhere | 481（54.54） | 416（51.11） | 347（50.14） | 51（56.04） | 1295（52.24） |
|  | Family sized cheese, cheese slices, etc. | 302（34.24） | 288（35.38） | 226（32.61） | 20（21.98） | 836（33.71） |
|  | Healthy dairy products suitable for serving with diets and wine, such as instant cheese | 194（21.99） | 170（20.89） | 177（25.54） | 11（12.09） | 552（22.26） |
|  | Instant desserts free from baking | 220（24.94） | 174（21.38） | 144（20.78） | 17（18.68） | 555（22.38） |
|  | Dairy products with high protein after body-building event | 116（13.15） | 88（10.81） | 99（14.29） | 12（13.19） | 315（12.70） |
|  | Prefabricated semi-dairy products that can be eaten by simple cooking | 135（15.31） | 123（15.11） | 94（13.56） | 11（12.09） | 363（14.64） |
