# Supplementary material for: Chinese residents’ knowledge about and behavior towards dairy products: a cross-sectional study
Source: BMC Public Health. 2023 Feb 21;23:374. doi: 10.1186/s12889-023-15254-1 (PMC9943042; doi:10.1186/s12889-023-15254-1)
Supplement: Supplementary file 4 — Additional file 4. Questionnaire on public milk drinking. [file 12889_2023_15254_MOESM4_ESM.docx]

**Questionnaire on public milk drinking**

Dear Ms. XX / Mr. XX,

In order to discover Chinese residents’ knowledge about and behavior towards dairy products and their influencing factors, as an attempt to lay a scientific basis for guiding Chinese residents rational intake of dairy products, we carried out this online survey in major cities around China. You are randomly selected as our interviewee, and your views and opinions are very important to us. We promise to keep your personal information strictly confidential. The contents of the interview are only used for this research, which will never be disclosed to any organization or individual without your permission. Thank you for your cooperation!

## 1. What is your gender? [Single choice]

| Male | 1 |
| --- | --- |
| Female | 2 |

1. **May I know your age please? ______ [Fill in the blanks]**
2. **What is your highest education level? [Single choice]**

| below undergraduate | 1 |
| --- | --- |
| Bachelor degree or above | 2 |

1. **Where do you live? [Single choice]**

| City | 1 |
| --- | --- |
| County | 2 |
| Countryside | 3 |

1. **Which of the following is your annual income level? The income here includes your salary, bonus, [ancillary](javascript:;) [revenue](javascript:;), investment income, pension and other income. [Single choice]**

| ＜50,000 RMB | 1 |
| --- | --- |
| 50,000-150,000 RMB | 2 |
| >150,000 RMB | 3 |

## **Do other family members living with you (including children over 3 years old) have the habit of drinking milk? [Single choice]**

| Yes, all of them | 1 |
| --- | --- |
| Yes, some of them | 2 |
| None of the them | 3 |
| I live alone | 4 |

## Lactose intolerance refers to the inability to completely digest and decompose lactose in dairy products, and diarrhea and other gastrointestinal discomfort symptoms may occur after having dairy products. Do you have lactose intolerance?[Single choice]

| Yes | 1 |
| --- | --- |
| No | 2 |
| Unknown | 3 |

1. **Which of the following do you think is dairy? [Multiple choice]**

| Milk | 1 |
| --- | --- |
| Yogurt | 2 |
| Milk powder | 3 |
| Condensed milk | 4 |
| Cream | 5 |
| Cheese | 6 |
| Food made of milk/milk powder as the main ingredient, such as milk flakes | 7 |
| Butter | 8 |

1. **Which of the following is more consistent with your understanding of milk?[Single choice]**

| Milk is a nutrient used to replenish the body | 1 |
| --- | --- |
| Milk is a daily necessity food as well as vegetables and fruits | 2 |

1. **What nutrients do you think you can get from milk?[Multiple choice]**

| Protein | 1 |
| --- | --- |
| Carbohydrates | 2 |
| Calcium | 3 |
| Fat | 4 |
| Vitamin | 5 |
| Inorganic salt | 6 |

1. **Do you think drinking milk is beneficial?[Single choice]**

| Very beneficial | 1 |
| --- | --- |
| There are some benefits | 2 |
| No benefits | 3 |

1. **What do you think are the benefits of drinking milk?[Multiple choice]**

| Improve immunity | 1 |
| --- | --- |
| Prevention of osteoporosis | 2 |
| Replenish energy | 3 |
| Calm the mind | 4 |
| Improve eyesight | 5 |

1. **What kind of dairy is recommended for people with lactose intolerance?[Single choice]**

| Fresh milk | 1 |
| --- | --- |
| Yogurt | 2 |
| Milk powder | 3 |
| Lactose free or low lactose dairy products | 4 |
| Can not eat any dairy products | 5 |

1. **Do you think people should drink milk every day?[Single choice]**

| Yes | 1 |
| --- | --- |
| No | 2 |
| It doesn't matter whether people drink milk or not | 3 |

1. **Which of the following people do you think should drink milk every day?[Multiple choice]**

| infants | 1 |
| --- | --- |
| children | 2 |
| teenagers | 3 |
| young people | 4 |
| middle-aged people | 5 |
| the elderly | 6 |

1. **How much milk do you think adult should drink per day on the average?[Single choice]**

| 100ml | 1 |
| --- | --- |
| 200ml | 2 |
| 300ml | 3 |
| 400ml | 4 |
| 500ml | 5 |

1. **At least how much calcium do you think an adult should take per day?[Single choice]**

| 400mg | 1 |
| --- | --- |
| 500mg | 2 |
| 600mg | 3 |
| 700mg | 4 |
| 800mg | 5 |

## How much milk, yogurt, milk powder or other dairy products do you consume on the average every day? (1 kilogram of cheese is approximately equal to 10 kilograms of milk) [Please fill in an integer, value > 0]_______ ML

## Generally speaking, how many days per week do you consume dairy products (such as milk, yogurt, milk powder, cheese, etc.) on average? [Please fill in 0 if you do not consume dairy products. Please fill in an integer with value range of 0 - 7]____ days

## How many years have you been taking dairy products? [Please fill in the value, keep at most one decimal place, the value > 0]_______years

## How often do you check ingredient statement on the label when you buy dairy products?[Single choice]

| Every time | 1 |
| --- | --- |
| Most of the time | 2 |
| Occasionally | 3 |
| Never | 4 |

1. **What do you want to know about dairy products？[You can select 2 options]**

| nutrition composition and function of dairy products | 1 |
| --- | --- |
| Production process and food safety control of dairy products | 2 |
| How to eat dairy products and pair them with other meals | 3 |
| How to make food from dairy products, such as homemade milk tea | 4 |
| How to choose the appropriate dairy products | 5 |
| How to solve problems related to dairy intake, such as lactose intolerance | 6 |

1. **In general, what do you value most when buying dairy products?[You can select 3 options]**

| Low fat/no fat | 1 |
| --- | --- |
| Low glucose/no sugar | 2 |
| High fiber | 3 |
| Added probiotics such as active lactic acid bacteria | 4 |
| Added minerals such as calcium, iron, zinc and selenium | 5 |
| Added vitamins | 6 |
| Added lactoferrin/whey protein | 7 |

1. **Which of the following possible additional consumption pattern of dairy products would you like to try in the near future？[You can select 2 options]**

| Dairy products in small packages that can be eaten anytime and anywhere | 1 |
| --- | --- |
| Family sized cheese, cheese slices, etc. | 2 |
| Healthy dairy products suitable for serving with diets and wine, such as instant cheese | 3 |
| Instant desserts free from baking | 4 |
| Dairy products with high protein after body-building event | 5 |
| Prefabricated semi-dairy products that can be eaten by simple cooking | 6 |
